# Providing spatial support during a major cholera outbreak in Port-au-Prince, Haiti: Creative mapping solutions in a challenging data poor environment

**DOI:** 10.1016/j.sste.2025.100753

**Published:** 2025-09-06

**Authors:** Andrew Curtis, Jayakrishnan Ajayakumar, Rigan Louis, Vanessa Rouzier, J Glenn Morris

**Affiliations:** a GIS Health & Hazards Lab (WG 82E), Department of Population and Quantitative Health Sciences, Case Western Reserve University School of Medicine, 10900 Euclid Avenue (WG Bldg Room 82F), Cleveland, OH 44106, USA; b Emerging Pathogens Institute, University of Florida, Gainesville, FL 32601, USA; c College of Nursing, University of Florida, Gainesville, FL 32601, USA; d Les Centres GHESKIO, Port-au-Prince, Haiti

**Keywords:** Grid heat map, Epidemic response, Cholera, Haiti

## Abstract

In this paper we describe the spatial data challenges faced in terms of providing accurate and timely analysis for a clinic during a cholera epidemic that spread through Port au Prince, Haiti in late 2022. This “triage” spatial epidemiology involved developing a bespoke geocoder that allowed for weekly maps of spread to be created in near real time. Resulting case data were also analyzed using a novel grid heatmapping approach which considers the epidemiological curve for each neighborhood. Adding further complexity during this period to both the data generation, and explaining cholera amplification and spread patterns, was a rising gang presence in the Port au Prince neighborhoods. Results identify a coastal pattern of amplification, which is expected given the informal settlement style living environments found in many of these neighborhoods. A second pattern then emerges of spread along a western and southern axis, which is far better captured in the grid heat mapping approach because of the lower numbers of patients seeking care at the clinic. The combination of traditional cartography and grid heat mapping help reveal the overall pattern of the epidemic, while also identifying key neighborhoods that require additional epidemiological investigation. Knowing why these neighborhoods played such an important role, possibly due to specific gang activity, is important in terms of understanding future disease spread in and around Port au Prince. Indeed, results presented can help contextualize official cholera reporting in 2025 where data availability is still hampered by ongoing gang rule.

## 1. Introduction

Any infectious disease outbreak in a city generating hundreds of cases can stress existing surveillance and epidemiological resources. This situation poses even more of a challenge in a data-poor environment such as Haiti. This is especially true for a cholera outbreak which often coincides with other system-wide shocks, such as a natural disaster, conflict, or other forms of violence. As is often the case, the spatial epidemiological response to such a cholera outbreak in an already data challenged environment becomes an exercise in resource-fulness, drawing on new and existing techniques to maximize insight from any available data, and if possible, providing near-real time guidance for responding entities. In this paper we detail that type of operation-styled response process used in Port au Prince (PAP), Haiti, during the cholera outbreak of late 2022.

Cholera remains a constant public health threat in environments lacking clean water and adequate sanitation (Morris, 2011). Simply put, water containing the bacteria, if ingested, can cause infection. From a clinical perspective, excessive diarrhea (sometimes over 1 liter/h in the most severe cases) can lead to dehydration, circulatory collapse, and death. The mortality rate in untreated patients can be up to 40%. It is therefore unsurprising that cholera outbreaks are often tied to societal disruptions, whether caused by natural hazards, political conflict, or violence. Most recently the conflict in Yemen (Camacho et al., 2018), and Sudan (Hassan et al., 2025), as well as the displacement of peoples in the Democratic Republic of Congo (Kayembe et al., 2023; Ingelbeen et al., 2019; Al-Rabeeah et al., 2025; Maisha et al., 2025; Ntumba Kayembe et al., 2025), have all been described in terms of cholera outcomes. Haiti has previously suffered similar complexities in terms of natural disasters, political upheaval and violence being attributed to causing, or exacerbating, cholera outbreaks (Etheart et al., 2018). One such external complexity, and a situation which continues to this day in Haiti, is a rise in gang violence that has massively disrupted society including its health response systems (Wadman, 2024). The challenge in a setting such as Haiti is how to identify emerging disease patterns given the obvious data constraints involved.

Historically Haiti was cholera free until a combination of the earthquake of January 2010, which impacted the country’s health infrastructure, and an external introduction of the disease by peace-keeping forces resulted in an epidemic with over 820,000 reported cases and close to 10,000 deaths (Mavian et al., 2022; Piarroux et al., 2011). The geographic pattern of this epidemic has been well studied with regards its origin and spread (Tuite et al., 2011; Page et al., 2015; Smallman-Raynor et al., 2015; Gaudart et al., 2013). However, as of February 2019, this epidemic was declared over with no further cases being reported. This situation changed in 2022.

There is some debate as to the permanent presence of cholera in Haiti, for example to what degree do environmental reservoirs play an ongoing circulating risk of infection (Mavian et al., 2022). However, if an enteric disease does emerge within or enter into an environment of densely packed dwellings, with inadequate clean water sources and no safe means of removing fecal matter, as is found in many PAP neighborhoods, an explosive amplification is always possible (Dunkle et al., 2011). Other contributing factors to the severity of such an outbreak include overall poor health (including high levels of stress), a lack of prior cholera vaccination, and limited access to health care (Piarroux et al., 2011). Adding further vulnerability in PAP was the rise in gang related violence during 2023-25, and the associated disruption of normal life and service provision, leading to the country’s overall descent into uncertainty (Olivier, 2023). While recent events have been captured in the media, the problems posed by gang violence in PAP reach back to before this period. For example, 71 people were killed by gangs in the PAP La Saline coastal neighborhood in 2018. One consequence of this violence has been the erosion of Haiti’s already fragile health sector and its ability to respond to significant events such as a disease outbreak (Rukovets, 2023). There have been various cartographic representations of the different gang territories^1^ in PAP, and while their control of the city in 2024 and 2025 was extensive, it is difficult to know how their growing presence was correlated to or causative of the late 2022 cholera outbreak. It is, however, extremely likely that gang activity, in combination with the other risk factors found in many PAP neighborhoods, make disease outbreaks more likely to occur (Larkin, 2023).

One example of how gang control has weakened local health response can be found with GHESKIO, a nonprofit organization that has been providing medical care primarily to low-income families in PAP and other parts of Haiti since 1982. Their main clinic and research center is situated on the Harry Truman boulevard, across the road from the Village de Dieu (from this point on VDV) neighborhood from where many of its patients originate. VDV, like many of the coastal neighborhoods includes densely packed informal settlement style living conditions. Many of these coastal neighborhoods emerged or expanded as part of the population relocation associated with the 2010 earthquake (Piarroux et al., 2022). VDV is also home to one of the most notorious gangs in Haiti.

As conditions in these neighborhoods make them vulnerable for a diarrheal disease outbreak, our team, in collaboration with GHESKIO, had been collecting fecal coliform samples at water points and in drainage locations to monitor the potential risks for residents (Curtis et al., 2019; Ajayakumar et al., 2022). Results revealed a highly complex environment where risk was considerable, and would vary by place and time, even within short geographic distances. Unfortunately, this monitoring stopped when the rise in local gangs made on-the-ground sample collection too dangerous for field teams, just as it did for many other health care services (Rukovets, 2023). As a result this form of ongoing cholera surveillance was ended because of gang presence. Other collaborative projects with the clinic also involved identifying local environmental cholera reservoirs (Mavian et al., 2022; Mavian et al., 2023; Paisie et al., 2023; Mavian et al., 2020), and the intersection of household behavior and typical Water Sanitation and Hygiene (WaSH) risks. While these continued in other neighborhoods, especially beyond the PAP area, eventually all these projects had been severely disrupted because of the widespread gang violence.

Despite recurrent political turmoil, persistent socioeconomic crises, gang turmoil and natural disasters, GHESKIO still managed to maintain its official role of outbreak surveillance and medical response for its surrounding area. There also remained an understanding that our spatial team would provide technical support to the GHESKIO Cholera Treatment Center (CTC) whenever it was needed. Such an event occurred in the middle of September 2022 when cholera cases started to rise in the PAP region. The number of cholera cases rose dramatically through October and into the first few weeks of November before eventually plateauing in December and declining in January (see https://mspp.gouv.ht/site/downloads/Sitrep%20cholera_11_F%C3%A9vrier%202023.pdf).

Mavian et al have previously provided a summary of the epidemic around GHESKIO (Mavian et al., 2023) as part of an investigation into the ancestral origin and diffusion of Toxigenic *Vibrio cholerae*. In their paper it was noted that the coastal neighborhoods, especially Waaf Jeremie (WJ from this point on) appeared to play an important role in the epidemic in terms of the patients seen at GHESKIO. The WJ neighborhood, just like VDD, can also be described as a coastal informal settlement. WJ was also under gang control and would eventually become part of the G9 gang coalition. La Saline, the previously mentioned location of the gang killings in 2018, lies between WJ and GHESKIO.

In this paper we expand on the work of Mavian et al describing the spatial data science methods developed to provide spatial support for the CTC, and presenting a more detailed geographic analysis of the cholera epidemic during its height between October and November 2022. We will show how spatial epidemiological tools in these settings must be nimble when analyzing spotty data streams for near real time insights. Advances include a new near real time surveillance geocoding system for the CTC and then the analysis of local patterns with a novel linked grid and cartographic heat map approach designed to understand the disease situation in the different neighborhoods, while also accounting for a distance-to-clinic bias. In 2025 PAP still remains largely inaccessible due to the ongoing gang activity (Harold Isaac, 2024), however the methods and results described in this paper can help shed light on the complex interaction between cholera spread and violence in the region, while also helping to contextualize the ongoing but sporadic cholera data briefs being released by the Haitian government.

## 2. Methods: triage spatial epidemiology

### 2.1. Solving a geocoding problem

**Fig. 1.**
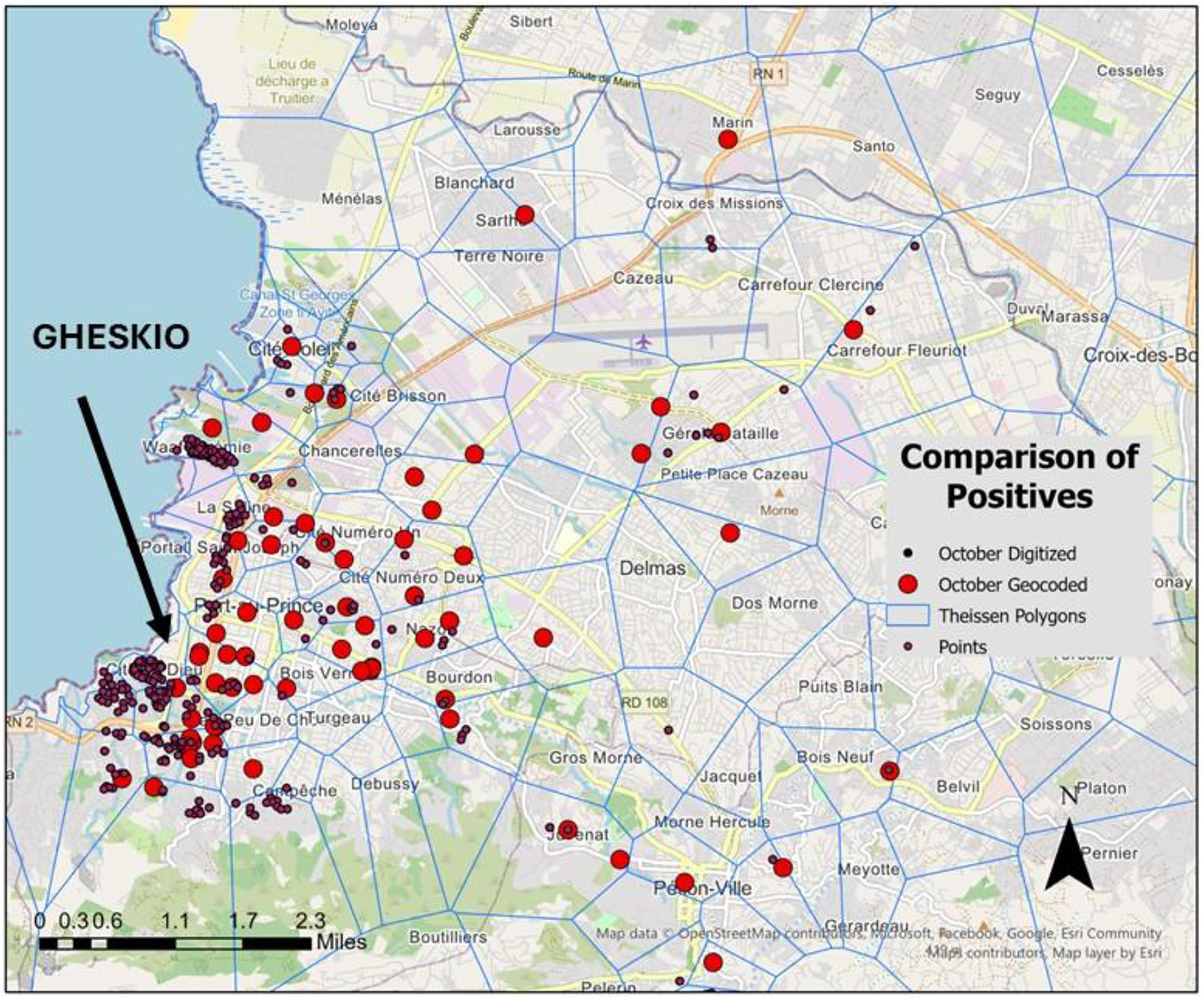
Initial geocode of suspected cholera cases supplied by GHESKIO. All of these locations have little on-the-ground precision and so therefore do not violate traditional spatial confidentiality mapping etiquette (Curtis et al., 2006).

During the early stages of the epidemic GHESKIO needed geospatial support to understand the geographic patterns of the cases it was treating. It is generally recognized that geocoding in Haiti is fraught with problems, especially at a granularity appropriate to support on-the-ground quality of care efforts (Griffiths et al., 2021). To this end de-identified cholera case locations were supplied by GHESKIO and then mapped using Google Maps. This resulted in a series of locations scattered across PAP (large red dots in Fig. 1).

Based on our mapping knowledge of the area, the lack of concentration towards the coastal neighborhoods was evidence of a geocoding problem. A deeper investigation of the supplied addresses found numerous geocoding errors, some of which have been previously identified in cholera studies for Haiti (Griffiths et al., 2021). PAP has virtually no standard geocodable address system (roads with house numbers). As a result only a few of the reported cases had typical addresses. Even if a street address was included, there was a high degree of uncertainty in location in terms of how that could be mapped as there was no commonly available household coordinate coverage, nor a digital street file with street segment address ranges. Simply put, for many neighborhoods in the coastal areas served by GHESKIO, “normal” street addresses did not exist. A second problem was that the spelling of place names often varied. Haitian French often uses phonetic spelling which means the written address can vary based on individual pronunciation. For example, WJ had the following variations (we are not including obvious spelling mistakes) Waaf Jeremie, Waffe Jeremie, Waff Jeremie, Waf Jeremie, and adding further error, Village Jeremie. There was also a disconnect between local knowledge (named areas and neighborhoods) and what appears on digital maps. For example, the map presented in McNairy et al shows the service region around GHESKIO and includes neighborhood names (especially Village de Dieu and Cite Plus), that are well known locally but do not appear on any of the typical mapping engine base maps (McNairy et al., 2019). A fourth more common aggregation problem is if an address is listed to the centroid of the “most likely” area it leads to artificial hot spots. This error was made more problematic in PAP as the location was often not even mapped to the correct general area. When the case was correctly mapped to the right administrative unit, the commune, the granularity needed to understand how different neighborhoods influence disease spread was lost. For example, the importance of the WJ neighborhood will be missed if cases were mapped to the centroid of the Cite Soleil commune which contains multiple neighborhoods and gangs. An official epidemic account, and popular media stories, describing the intensity of disease in PAP often refer to Cite Soleil. The importance of the WJ neighborhood would not be mentioned. While any normal batch address match must deal with variations of all these problems, they usually, in total, present minimal additional effect in terms of correction, which from our experience involves no more than 20% of all geocodes. This means in a normal geocoding situation, a good faith effort in identifying and rectifying the problem addresses will lead to an acceptable (and hopefully unbiased) mapped output. The difference with the cholera case data supplied by GHESKIO was that arguably every address had to be questioned. This also had to happen in near real time as the epidemic unfolded. Added to this was a high degree of stress on the data producing side where employees were working in epidemic conditions while also being proximate to an area of high gang activity. The GHESKIO employees also had little guidance in terms of how to record addresses correctly, which again introduced an understandable amount of human error in the data coming from the CTC.

To counter these geocoding challenges, each cholera address in the beginning of the epidemic was digitized into Google Earth manually using various map sources as guides, including Google Earth imagery, online OpenStreetMap, Arc GIS base imagery (multiple sources), and recent academic publications and websites. As a result, 312 addresses were mapped to an acceptable level of precision. While some of these addresses remained as polygon centers, considerable improvement in the accuracy of the geocode was achieved. Systematically “learning” from the way the addresses were written, and then mapped, a process which also included feedback with our Haiti collaborators in determining their intent when writing certain place names (such as Cite Soleil), led to a more appropriate distribution of suspected cases (smaller dots on Fig. 1) that better matched our local understanding of the most vulnerable neighborhoods. It is important to note that these addresses still could not be precisely mapped due to the lack of street names and addresses but rather the cases are located using informed guesses based on high resolution aerial imagery or “fuzzy placement” which utilizes a hierarchical space filling approach we had developed for Haiti-like environments.

While our mapping team had not worked explicitly in WJ, it had collaborated with GESHKIO extensively in Cite Plus and VDV until gang activity had halted studies. The ariel imagery of WJ suggested a similar density and type of residence as VDV. This similarity was confirmed by the GHESKIO team. It was therefore decided that a hierarchical space-filling approach to digitize locations would be appropriate. This approach starts with placing a case at the centroid of the polygon (such as a neighborhood). Subsequent cases are then located in the largest remaining gaps, resulting in the available spaces continually being reduced in size. Similarly, for roads, the first case would be placed in the middle of the road segment, with subsequent cases then dissecting the remaining road lengths available. In both these examples, on-the-ground information and imagery would also inform the choice, for example gaps would be filled first where there was a higher density of structures, while areas of non-habitation, such as a water body, would not be chosen. While not precise, this method still captures the accuracy of being in the right neighborhood but does not over saturate a single location with a false precision.

The resulting PAP maps were frequently sent back to the GHESKIO team for additional local insight regarding the choice of names, where best to place cases in “fuzzy” locations, such as along a long street with commercial sections, or when a place name with no obvious residences had been chosen. This type of participatory mapping utilizing feedback is not uncommon for research in Haiti (Griffiths et al., 2021). This entire process occurred over a matter of days and resulted in a more robust standardized approach for the data coming from GHESKIO for the rest of the epidemic. This was vital in being able to produce more effective near real time maps of disease spread, while still not being overly arduous on the clinic’s data team.

A traditional graduated color mapping approach was also developed to produce a more conservative aggregation surface based around known neighborhood names. This approach both limited the impact of the “fuzzy” case placements by aggregating to more robust place names, while still providing more granularity than the normal administrative boundaries used by the Ministere de la Sante Publique et de la Population (MSPP) to “officially” map the progression of the epidemic, and which failed to capture the detail needed to understand local disease spread in the neighborhoods around the clinic (Griffiths et al., 2021). The earlier mentioned problem of WJ being assigned to Cite Soleil was an example of this aggregation problem. As an alternative mapping strategy every known neighborhood or settlement in the PAP area was represented by a centroid at its most representative place on the map. Some of these neighborhoods were found on the previously mentioned digital map sources, others came from online sources and academic publications, and a third group involved local neighborhood names that had to be checked with the GHESKIO team to determine location. Once an exhaustive list of neighborhoods was mapped, a Thiessen Polygon (TP), also known as a Voronoi surface, was created to form a new polygon map layer. TPs equally divide space according to key nodes (neighborhood centroids) which means that every part of the map is assigned to a polygon which contains its closest neighborhood centroid (Fig. 1 contains the TP outlines). This approach has previously been used by our team in health challenged environments with a lack of established neighborhood area boundaries (Bempah et al., 2020; Bempah et al., 2022) as well as by Griffiths et al in analyzing cholera in one rural Haitian province (Griffiths et al., 2021). While the outer polygon shapes might not follow neighborhood boundaries (such as a peninsula) or can be distorted because of no outer extent, it still provides a better mapping scheme for the central areas of PAP, especially around GHESKIO, which was the geographic focus. The natural form of the coastal neighborhoods, which include many of the most vulnerable and densely populated areas in the city, are well captured using this approach. While additional accuracy could be achieved by manually improving boundaries, those used in this paper are the same as those mapped during the epidemic response.

**Fig. 2.**
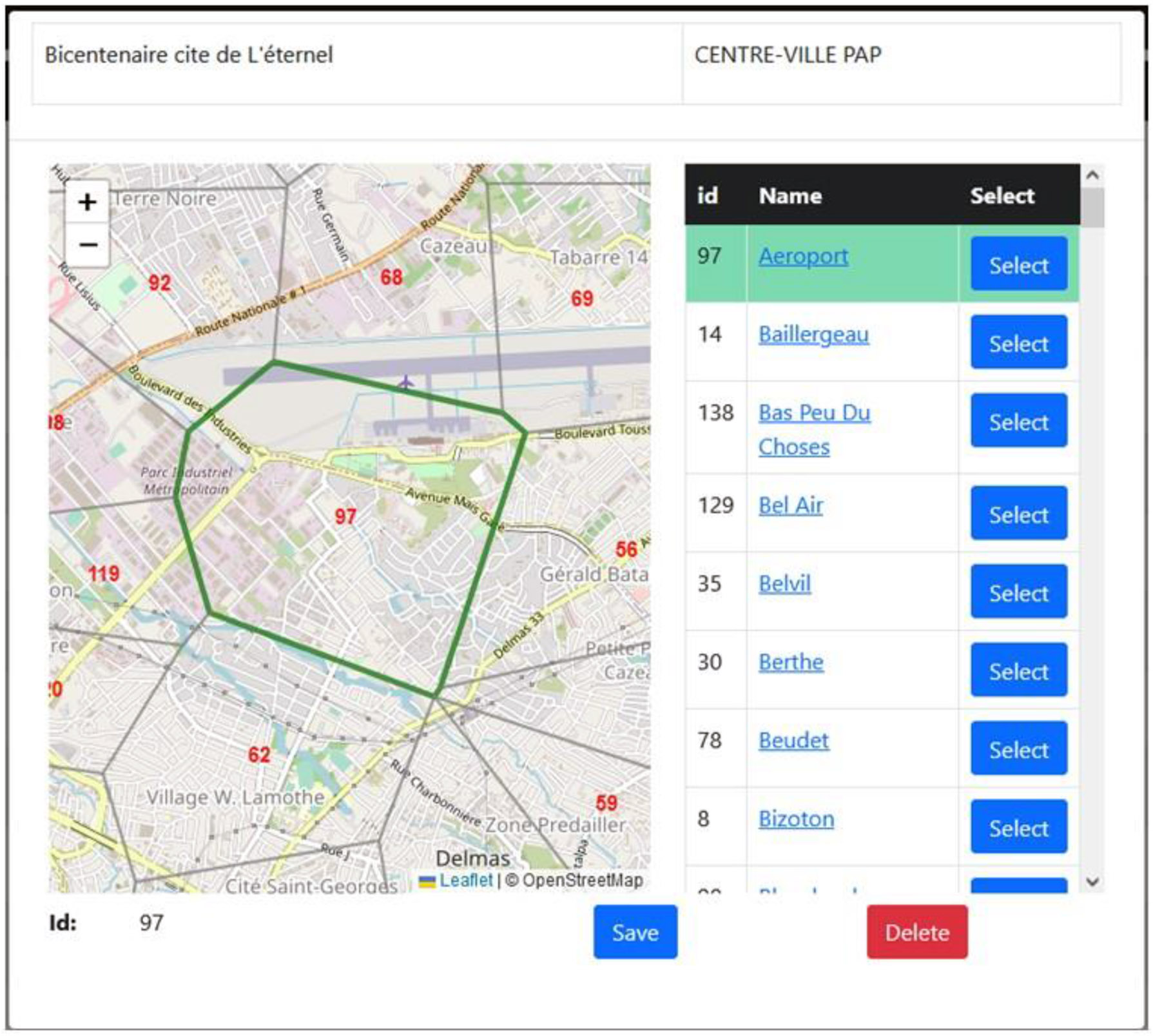
The geocoder developed for GHESKIO to support their suspected cholera case uploads. The geographic tagging of neighborhoods could be achieved using name matching or by identifying and clicking the correct place on the searchable map.

Once the TP layer was created, a bespoke web-based geocoder was developed to support the GHESKIO team at data entry. This allowed the data team in Haiti to spatially tag each case to a specific TP either by browsing the list of neighborhood names or by interacting with the map and selecting the right area. This approach also allowed for more local knowledge or insight to guide where a case should be located (Fig. 2).

Once completed the new spreadsheet, with the polygon names added, was emailed to the North American team who joined the data to the TP map layer. These data were investigated for spatial trends in the outbreak in near real time, including which neighborhoods had the most disease, and how the situation around GHESKIO was changing.

### 2.2. An epidemiological curve heat map

Exploratory data analysis of space-time patterns can be challenging for more granular situations (AvRuskin et al., 2004). Typically a geocode, such as a neighborhood, occupies one axis, and time forms the second axis. Space-time analytical techniques often involve threshold levels where an interaction exceeds an acceptable probability (Kulldorff, 1997). While this approach can reveal overall patterns at the end of an epidemic, for example where one area had a significant rise in cases during one period, it is less useful in extracting more complex dynamically changing patterns. One solution for operations based epidemic mapping is to instead consider a multiple epidemiological curve approach for a region. Conceptually this means each neighborhood can be visualized in terms of when cases rise, plateau and drop, with data being added on a weekly basis. By comparing these curves, broader regional trends can also be identified.

**Fig. 3.**
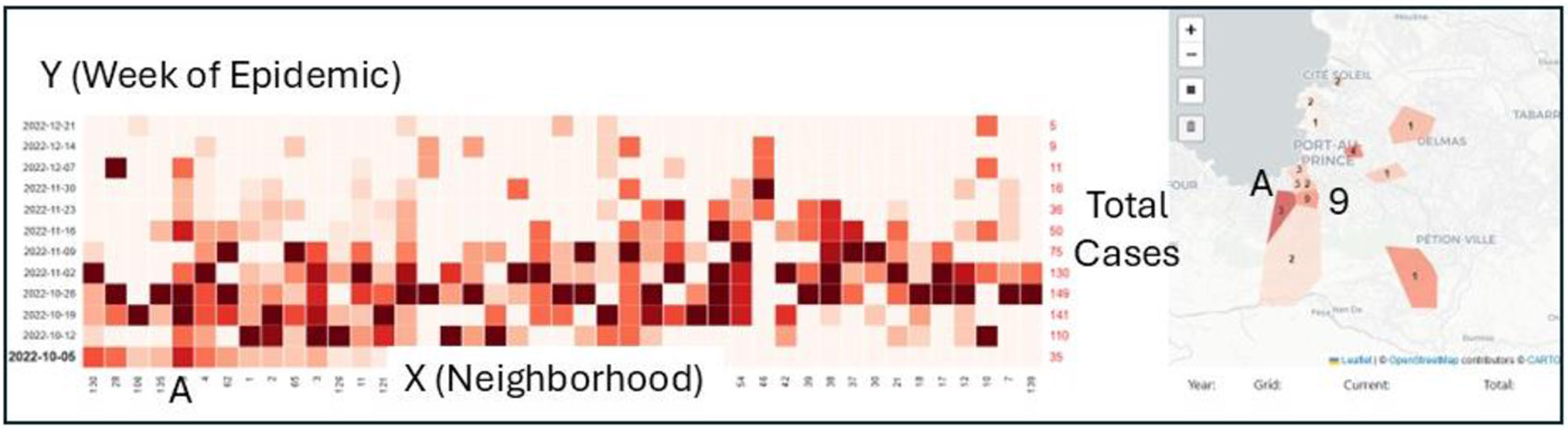
An example of the combined heat map and cartographic display for the first week of this epidemic.

To achieve this we adopted a version of a grid heat map (Gu, 2022). Simply put, each geocode column (x axis) contains temporal cells (y axis), in this case each week of the epidemic (Fig. 3). The week with the highest number of cases receives the darkest shading in the temporal column. All other cells in that column are then normalized as a proportion of that maximum disease count. The finished heat map allows for a visual investigation of where the location-standardized case intensities occur. Conceptually it can be considered as a visualization of the different neighborhood epidemiological curves, and as such can capture more subtle trends within lower case number areas.

To achieve this a combined grid and cartographic heatmap interface was programmed in Python that displayed weekly aggregations of case counts for each TP on the x axis, and the week of the epidemic on the y axis. Connected to this grid is a hyperlinked cartographic display meaning that if a particular week was selected, then the cartographic map would display the same TP coloring as seen in the grid. Clicking on any cell would also highlight the appropriate TP on the cartographic map, thus facilitating the exploratory investigation of space and time patterns using both types of heatmaps (Fig. 3). For example, neighborhood A (which is a single TP) in Fig. 3 displays a typical epidemiological curve, with a ramp up of cases until the third and fourth week of the epidemic, and then a relatively gradual decline. All other weeks for this neighborhood are colored according to their proportion of the case total for week 3 and 4. The bolded date on the left of the grid is the week being considered, and the neighborhoods are shifted on the Y axis based on their similarity, which basically means those with their highest number of cases during that week are gathered. The map is also interactively linked to the grid heat map based on the week being displayed. In Fig. 3 we can see that the intensity for neighborhood A is one of the darkest browns. However, in the grid heat map, for that neighborhood there is another week (3 and 4) that has more cases. This means on the cartographic map neighborhood A has a darker shade but still not it’s darkest color. There are also other neighborhoods with lighter colors than A on this map but with more cases, such as Bolosse with 9 (the total number of cases are displayed for each neighborhood TP). While this is counter intuitive for traditional choropleth mapping, it does provide a novel way of visualizing how the increases in cases for any neighborhood form part of a broader pattern or trend, rather than just displaying where the most cases are each week. This is particularly insightful if there is a bias in the data, such as a distance decay effect associated with TPs further from GHESKIO, or if there are (unknown) variations in numerator populations. Unlike other techniques, these low numbers are still important to understand the less data rich geographies of the epidemic. Finally, the numbers in red on the right Y axis are the total number of new cases occurring each week.

## 3. Results

A total of 860 suspected cholera cases were reported by GHESKIO for October (447) November (361) and December (51). While most cases had been geographically stamped using the new geocoder beginning in November, each address was still mapped out to a single location using the approaches previously described. Approximately 10% of the addresses were returned to the GHESKIO team for additional mapping guidance, for example, what was meant by “Bicentenaire” which does not appear on maps but is a relatively well-known local name in PAP.

**Fig. 4.**
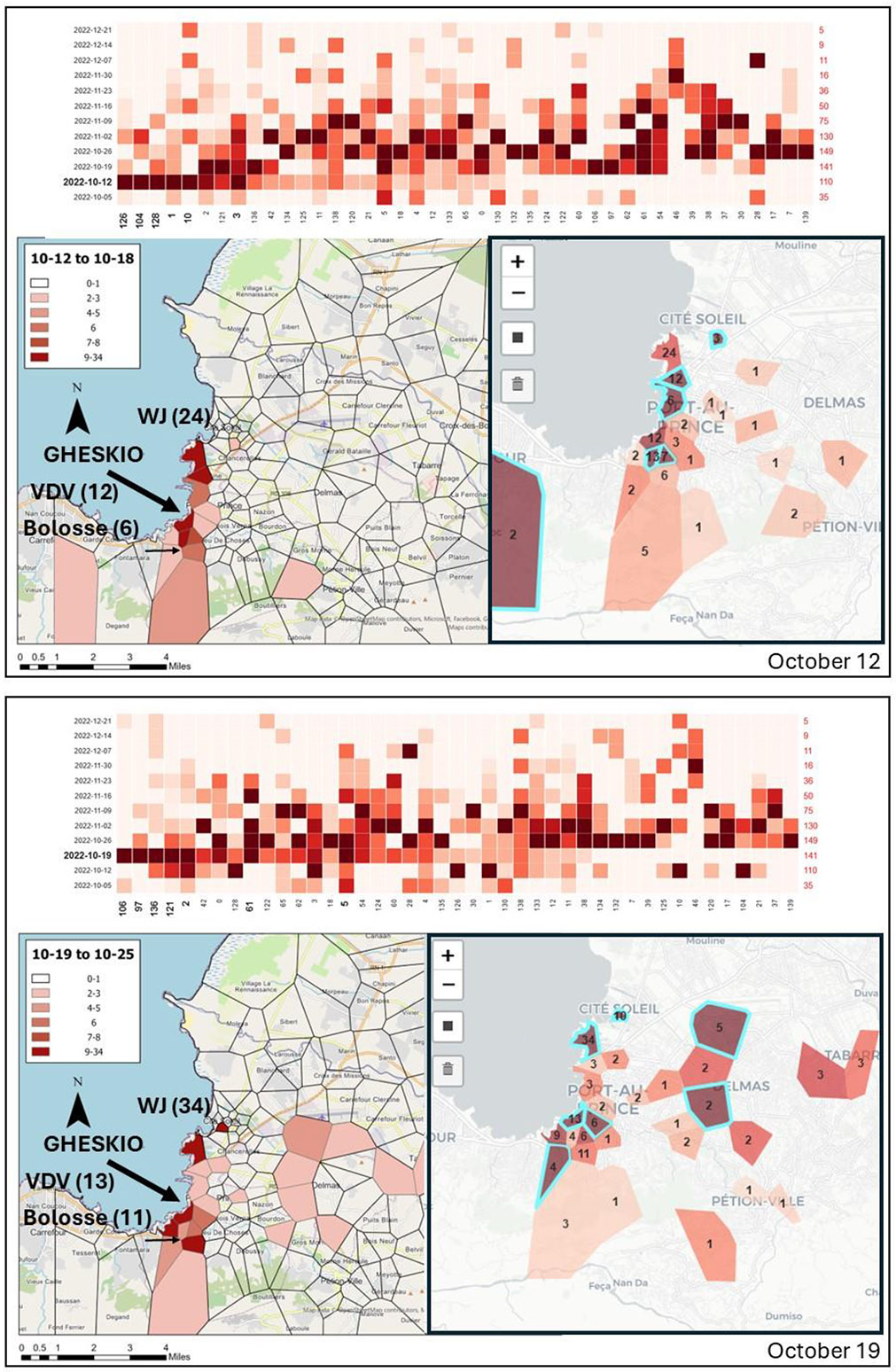

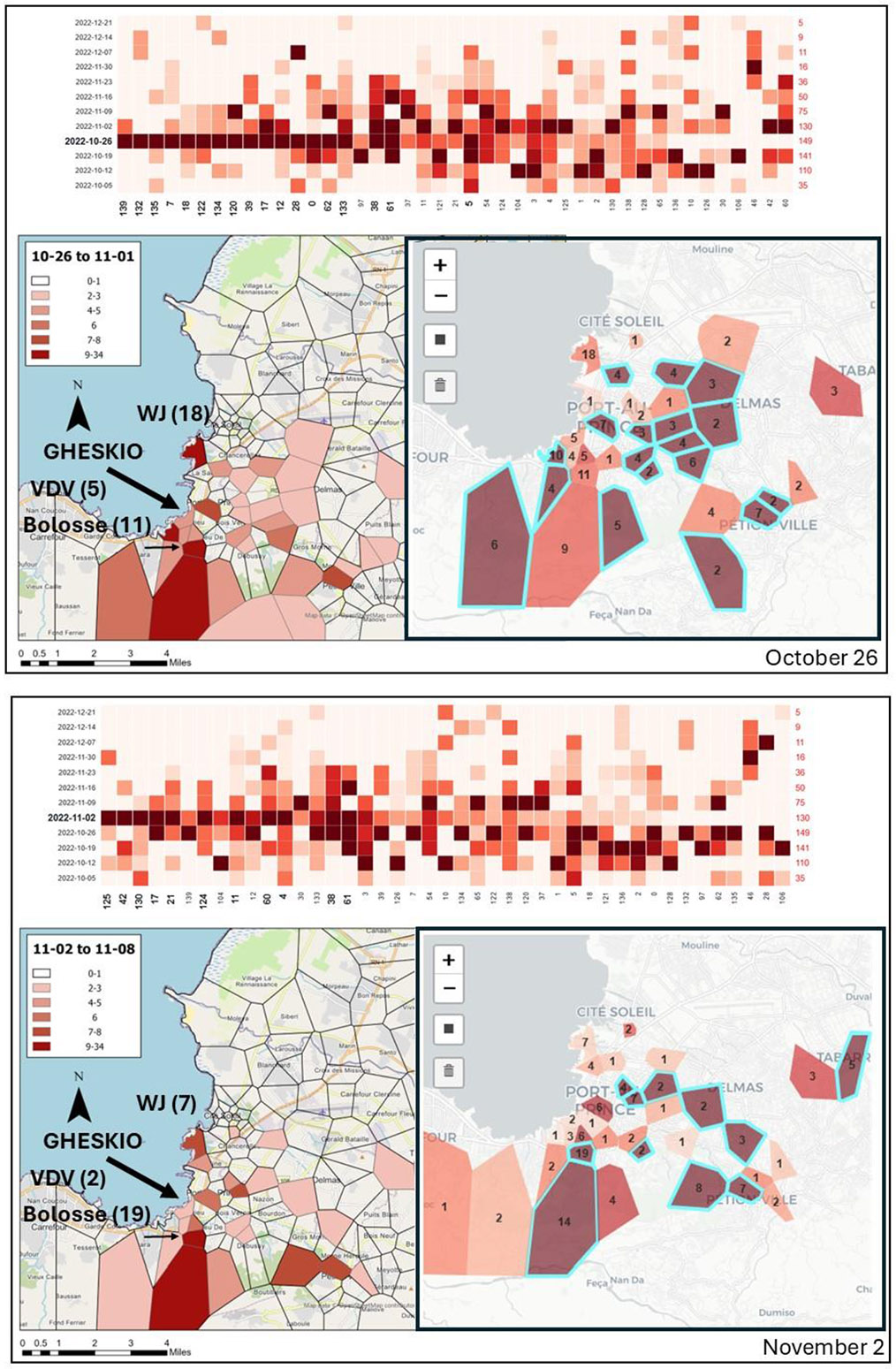
Comparing traditional graduated color mapping of suspected cholera cases with the grid heat map for the Thiessen Polygon geocodes for the four most important weeks of the epidemic (10/19, 10/26, 11/02, 11/09). The main map of each figure is a typical cartographic representation using the same class breaks across each time period. The inset map matches the colors of the grid heat map where every neighborhood is displayed as a proportion of its highest weekly total. For comparative purposes the weekly number of cases in the Waaf Jeremie, Village de Dieu and Bolosse neighborhoods are included. These grid heat maps were created once the epidemic had declined and the highest weekly total was known. During the epidemic, heat maps were dynamic with incoming weekly totals changing the pattern to help reveal neighborhood diffusion in near real time.

Once all addresses had been checked, cases were joined to the TP layer to create a series of weekly graduated color maps of patients attending GHESKIO visualized using the combined grid heat maps (Fig. 4).

For each week the most similar TPs are gathered towards the Y axis to help explain regional patterns. To further help with interpretation, those neighborhoods with the highest number of cases occurring in that week are highlighted with electric blue in the accompanying inset cartographic map. The numbers in red at the side of the heatmap show the total number of new cases seen at GHESKIO for that week. For the week beginning October 12^th^, the two coastal neighborhoods WJ and VDV, along with La Saline which is immediately south of WJ produce the most cases. The grid heat map cartographic display reveals a nuanced variation of the pattern, most of the other coastal neighborhoods report their highest disease total during this week. By contrast, WJ has not yet peaked. When we move to October 19^th^, a similar pattern emerges with many of the remaining coastal neighborhoods recording their highest weekly totals, including those closest to GHESKIO, such as VDV. There is also a slightly southern directionality to the increases in cases. The variation to this pattern is WJ to the north which also receives its highest number of cases during this week (34), which is a higher total than for any neighborhood during the epidemic. And yet the neighborhoods between WJ and GHESKIO appear to be relatively lightly impacted. Adding further mystery is the neighborhood to the northwest of WJ which also receives its highest total (10) during this week. On further investigation this neighborhood “Soleil” contains points that are probably assigned to the commune aggregation. As WJ is in this Cite Soleil commune, it is possible these should be added to the WJ total, but it is also possible they were a catch all for areas within Cite Soleil that were not WJ. A further pattern to notice is the westward spread of cases, with a few neighborhoods having their highest total during this week. However, it is the following week that even better captures this spread. While WJ has the highest total for the week (18), there appears to be an overall decline in the coastal neighborhoods. What is more apparent from the grid heat map is a southern and western spread with the majority of the neighborhoods receiving their highest total during this week. A pattern that is harder to distinguish in the more traditional cartographic representation. By the week starting November 2^nd^, this pattern has continued, with the coastal neighborhoods dropping considerably, including WJ, and with the western and southern axis intensifying, with many neighborhoods receiving their highest case totals, including Bolosse, and to the south west, Petionville.

In summary, both forms of mapping revealed a potential start to the epidemic south of GHESKIO, with amplification in the coastal neighborhoods, especially WJ. The grid heat map better captures the westward and southward diffusion axis as the coastal neighborhoods start to lose their intensity.

## 4. Discussion

Operations based spatial epidemiology, in this sense meaning the task of making sense of emerging patterns and trends as an infectious disease outbreak unfurls, is challenging in even the most data rich environments. In this paper we describe the spatial data problems faced by a clinic in Haiti operating in a data challenged environment during an ongoing cholera outbreak. In addition, this epidemic also coincided with a rising level of violence which would eventually lead to the majority of PAP falling under gang control. While the response and details of this epidemic may be unique to Haiti, the challenges faced, and the creative solutions needed, are probably typical of many other environments where cholera epidemics are likely to occur.

In 2022 Haiti was a country already stretched in terms of adequate medical resources and a societal hesitancy towards seeking medical care (Medozile et al., 2023). Making that situation even more dire was the ongoing political disruptions faced by the Haitian government and the subsequent rise to power of several neighborhood gangs. It is difficult to know how violence affects the epidemiology of an epidemic in terms of creating diffusion patterns within neighborhoods, across the city, and out into the broader region. It is also difficult to know how a combination of existing disease vulnerabilities in the coastal neighborhoods, in combination with the rise in gang presence, will have further exaggerated the spatial heterogeneity of disease amplification risk. Gang activity had restricted the routine operations of both private and public institutions, including the health sector such as the university of Haiti’s general hospital, and GHESKIO, during 2024 to 2025. To what degree had those processes already been causative or correlated to events in late 2022 is again hard to ascertain. Our own experience had seen a disruption in existing enteric disease surveillance programs for the coastal neighborhoods of PAP. A further horrendous illustration of the gang-disease intersection, and one that directly impacted the WJ neighborhood, occurred in December 2024 when 184 people, including many elderly, were executed for using “voodoo” to cause their leader’s son to fall sick and die (Blaise, 2024). How might have this same gang impacted disease amplification, spread, and care seeking in WJ during 2022?

Given such uncertainties, this paper focused on what was still possible with the available data. The spatial data science solutions described included a novel geocoder that helped support GHESKIO, producing data that could be used for situational mapping. The resulting granularity provided more appropriate on-the-ground detail than was available at the time through Haitian government reporting mechanisms. This geocoder, which initially combined local knowledge, existing map files, and geographic detective work to map out suspected case locations, used a Thiessen Polygon label approach to be incorporated as part of the daily recording process for the CTC. The quality of these data also allowed for a novel visualization approach which had previously been used by the team during Covid 19 to reveal neighborhood diffusion patterns in the United States (Curtis et al., 2022). This visualization mapped the geographic variations of all neighborhoods using their epidemic curves rather than more typical rate or count distributions. This form of mapping is not only useful to understand dynamic diffusion as the epidemic happens, it also helps extract more meaning from a geographically biased dataset, in this case influenced by proximity to GHESKIO.

Summarizing the epidemic, for the neighborhoods around GHESKIO, during the first week of the outbreak (10/05 and also seen in Fig. 3) there was little evidence of the coming importance of the coastal areas. Indeed, the neighborhood with the highest number of cases was Bolosse to the south with 9. The pattern for the second week of the epidemic (the week of October 12^th^) revealed the rise in cases in the informal coastal neighborhoods, many of which generated their highest number of cases at this point. Except that is, for WJ, which, though it had the highest numbers of cases of any neighborhood in this week (24), had still not reached its own epidemic apex which arrived the next week (the week of October 19th) with 34 new cases. VDD, the neighborhood opposite GHESKIO, also recorded its highest number of cases this week (13). Both neighborhoods are characterized by typical informal settlement conditions, so it is not surprising that when disease entered PAP these would be the sites of amplification. Though there were still geographic variation along the coast with the two neighborhoods situated between VDD and WJ, including La Saline, quickly tailing off.

Where the grid heat map provided a different insight was in interpreting the geographic pattern in the neighborhoods further away from GHESKIO, and from where there would be fewer expected patients. The weeks starting October 26^th^ and November 2^nd^ clearly show a spread away from the coast on both a southern and westward axis. While these numbers are lower, and therefore create a more muddled pattern in the normal cartographic representation, this diffusion is clearly seen in the grid heat map. Again, this is not an unexpected result, the conditions of the coastal neighborhoods are likely to result in an initial amplification of the disease, which would then spread outwards into the larger PAP area. What is somewhat surprising is the role of Bolosse, to the south, which records the highest number of weekly cases in both the first, and last week of the epidemic displayed here. It is likely that the number of cases seen by GHESKIO from this neighborhood is an undercount because of the distance to the clinic. By considering its epidemic curve we can see that there is a steadier increase in cases across the weeks, not as explosive as for the coastal neighborhoods, but eventually rising to its peak in the week of 11/02, which at this point even exceeds WJ in terms of new cases. This area generated the first cases and then outlasted the other neighborhoods. Was this area where the epidemic started? Were the more extreme epidemic curves in the coastal neighborhoods because of the poor local conditions and gang activity?

A second nuanced pattern emerges from the interior stretching southwest towards Petion Ville. Again, for these neighborhoods with relatively fewer patients visiting GHESKIO, the more traditional mapping pattern reveals less of a regional trend. Of course, any spatial signature should be treated extremely cautiously, especially as at the time of writing there is still little ability to communicate with GHESKIO because of the ongoing gang situation.

## 5. Conclusion

While most spatial epidemiology requires robust and reliable data to be analyzed with traditional techniques, from a response perspective making sense of disease patterns as they happen is vital even in data poor environments. To achieve this requires a nimbler and more creative mindset (Curtis et al., 2022). Two examples of this triage mentality during the cholera epidemic of 2022 included improving geocoding and then using a novel form of heat mapping to monitor individual neighborhood disease progression. It was hoped that this new geocoding approach could provide an ongoing means for more granular insights in cholera intervention in Haiti (Griffiths et al., 2021). It was also hoped a version of this system could evolve further to create a new standardized geocoding approach at GHESKIO for all its health interactions. However, there has been little chance to further improve this system through evaluation and feedback because of the ongoing local gang activity.

The use of heatmaps did prove insightful for the type of data collected by the GHESKIO CTC, especially as they were at a spatial resolution not available through other official sources. Questions raised included how did disease initially enter PAP, then move between neighborhoods, and why was there such explosive growth in some areas, especially WJ? Most intriguing, how were many of these epidemiological questions changed by the local gang involvement? When PAP returns to normalcy, there should be a retrospective investigation to understand why this one coastal neighborhood was so important in the outbreak. Was the nature of the epidemic influenced more by site (local environmental conditions) or situation (the local specific gang involved) or a combination of both? The answer could help prevent the occurrence of similar epidemics in Haiti, as well as providing a methodological roadmap for cholera risk in other conflict dominated areas.

## Data Availability

The data that has been used is confidential.

## Footnotes

1 For example see https://globalinitiative.net/analysis/haiti-gangs-organized-crime/; https://i.redd.it/updated-gang-map-v0-urox5t3j4fd91.jpg?s=cb549c4b1dd850236891650169711b328c477c3a; https://www.google.com/url?sa=i&url=https%3A%2F%2Fglobalinitiative.net%2Fanalysis%2Fhaiti-gangs-organized-crime%2F&psig=AOvVaw1HCi0oFoZ8zreAm1VO2js7&ust=1684510459924000&source=images&cd=vfe&ved=0CBEQjhxqFwoTCMjit86Y__4CFQAAAAAdAAAAABAS; https://www.barrons.com/news/gang-activity-in-haiti-s-port-au-prince-5b763eaa
